# Activation of G Protein-Coupled Estrogen Receptor 1 (GPER) Attenuates Obesity-Induced Asthma by Switching M1 Macrophages to M2 Macrophages

**DOI:** 10.3390/ijms25179532

**Published:** 2024-09-02

**Authors:** So-Eun Son, Dong-Soon Im

**Affiliations:** 1 Department of Biomedical and Pharmaceutical Sciences, Graduate School, Kyung Hee University, Seoul 02447, Republic of Korea; seson@khu.ac.kr; 2 Department of Fundamental Pharmaceutical Sciences, Graduate School, Kyung Hee University, Seoul 02447, Republic of Korea

**Keywords:** GPER, GPR30, estrogen, obese, asthma, lung

## Abstract

The prevalence of obesity-induced asthma increases in women after menopause. We hypothesized that the increase in obese asthma in middle-aged women results from estrogen loss. In particular, we focused on the acute action of estrogen through the G protein-coupled estrogen receptor 1 (GPER), previously known as GPR30. We investigated whether GPER activation ameliorates obesity-induced asthma with a high-fat diet (HFD) using G-1, the GPER agonist, and G-36, the GPER antagonist. Administration of G-1 (0.5 mg/kg) suppressed HFD-induced airway hypersensitivity (AHR), and increased immune cell infiltration, whereas G-36 co-treatment blocked it. Histological analysis showed that G-1 treatment inhibited HFD-induced inflammation, fibrosis, and mucus hypersecretion in a GPER-dependent manner. G-1 inhibited the HFD-induced rise in the mRNA levels of pro-inflammatory cytokines in the gonadal white adipose tissue and lungs, whereas G-36 co-treatment reversed this effect. G-1 increased anti-inflammatory M2 macrophages and inhibited the HFD-induced rise in pro-inflammatory M1 macrophages in the lungs. In addition, G-1 treatment reversed the HFD-induced increase in leptin expression and decrease in adiponectin expression in the lungs and gonadal white adipose tissue. The results suggest that activation of GPER could be a therapeutic option for obesity-induced asthma.

## 1. Introduction

Increase in the prevalence of obesity has become a critical problem affecting the quality of life and health worldwide [1]. Obesity is now considered a driving force that exaggerates disease conditions by producing pro-inflammatory adipokines and cytokines [2,3]. Metaflammation, which is a chronic state of low-grade inflammation in the adipose tissues, may contribute to the development of airway inflammation, that is, obesity-induced asthma [4]. Elevated levels of interleukin-6 (IL-6) and tumor necrosis factor (TNF)-α in adipocytes are directly associated with production of IL-1β, IL-4, and IL-5 in the bronchial epithelium [2,3]. Decreased adiponectin and increased leptin have been connected with insulin resistance in obesity, along with changes in other inflammatory cytokines [5,6]. Leptin not only acts as a satiety signal in the hypothalamus but also stimulates the rise of IL-6 and TNF-α in adipocytes and increases expression of interferon (IFN)-γ by peripheral-blood mononuclear cells [7,8].

Epidemiological studies have suggested an association between sex hormones and the incidence of asthma [9]. Notably, the prevalence of asthma in boys is higher than in girls; however, it is more common in adult women than in men [10]. In normal-weight women, increasing years of pill use are associated with a reduced risk of current asthma [11]. In a large population study of adults from the National Health and Nutrition Examination Survey, higher levels of serum estradiol in obese women are associated with a 40% reduction in the odds of current asthma [12].

In premenopausal women, endogenous estrogens largely prevent the development of hypertension, dyslipidemia, and diabetes by inhibiting inflammation [13]. The effects of estrogen can be divided into acute and chronic [14]. Chronic and genomic effects of estrogens are largely evoked by the ER-α and ER-β nuclear estrogen receptors, which modulate the corresponding gene expression [4]. The acute and non-genomic effects of estrogens, such as rapid vascular effects, are mediated partly by G protein-coupled estrogen receptor 1 (GPER) [14,15], which was initially cloned as GPR30, an orphan receptor differentially expressed in estrogen-responsive breast cancer cell lines by multiple groups [16,17,18]. Notably, GPER is expressed in most organs, including the lungs, heart, brain, liver, kidneys, pancreas, and skeletal muscles [19]. Binding affinities of 17β-estradiol for ER-α/β and GPER were estimated to be in the range of 0.1–0.5 nM and 3–6 nM, respectively [14]. The pharmacological tools for GPER such as G-1, the highly selective GPER agonist, and G-15 and G-36, the GPER antagonists [20,21,22], which show no significant binding to nuclear ER-α/β, have accelerated in vivo studies of GPER in animals [10]. The GPER-dependent regulation of inflammation and lipid metabolism has been reported in studies on obesity, diabetes, and atherosclerosis. Furthermore, GPER-knockout mice develop visceral obesity [23,24,25]. Knockout of GPER gene makes mice be obese by rises of fat (perivascular, subcutaneous, and visceral) [23,24,25]. Additionally, GPER modulates immune responses, consistent with its anti-inflammatory role [23,25]. Considering that the prevalence of obesity-induced asthma increases in women after menopause, we hypothesized that estrogen loss may result in the rise in obese asthma in middle-age women. Considering the protective roles of estrogen in obesity and inflammation, we investigated whether GPER activation with G-1 could ameliorate high-fat diet (HFD)-induced obese asthma in combination with the GPER antagonist G-36.

## 2. Results

### 2.1. G-1 Improved the Glucose Tolerance Capacity; However, It Did Not Suppress the HFD-Induced Obesity in C57BL/6 Mice

**Figure 1:**
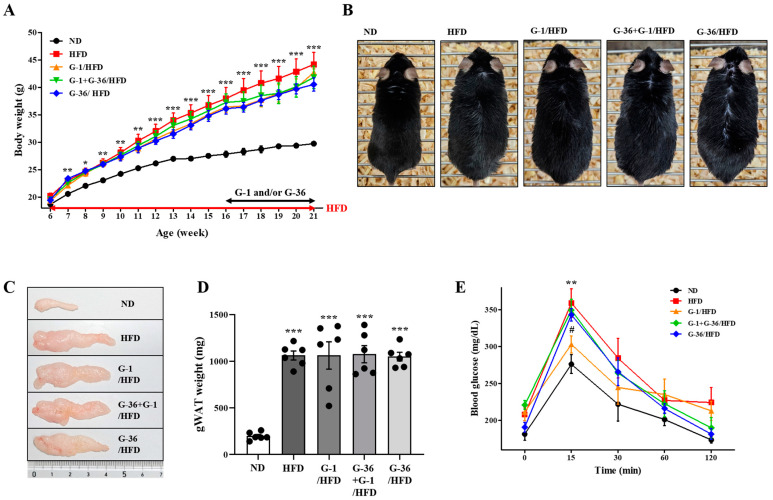
G-1 improved the HFD-induced glucose intolerance; however, it did not affect weight gain and adiposity in C57BL/6 mice. We assessed the effects of G-1 and/or G-36 on adiposity, glucose intolerance, and weight gain induced by HFD. (**A**) Changes of body weight. We fed mice an ND or an HFD from 6-week-old mice for 15 weeks. G-1 and/or G-36 was delivered by i.p. injection at a dose of 0.5 mg/kg from 16-week-old age for 5 weeks. Then, we euthanized the mice. (**B**) Photos of 21-week-old mice. (**C**) Photos of gonadal white adipose tissue. (**D**) Weight of the gonadal white adipose tissue from 21-week-old mice. (**E**) Oral glucose tolerance test was conducted in 21-week-old mice. * *p* < 0.05, ** *p* < 0.01, and *** *p* < 0.001 vs. the ND group, # *p* < 0.05 vs. the HFD group. Values are presented as the mean ± SEM of six mice.

As shown in Figure 1A, we fed 6-week-old male C57BL/6 mice with HFD for 15 weeks. The HFD induced a significant gain in body weight in male C57BL/6 mice than in normal diet (ND)-fed mice (Figure 1A). The body weight was increased about 45% by HFD compared to the ND group (Figure 1A). Mice were simultaneously treated with G-1 and/or G-36 (0.5 mg/kg, i.p., five times per week) from 16-week-old age for 5 weeks (Figure 1A). G-1 or G-36 administration did not cause any change in weight gain (Figure 1A,B). The weight of gonadal white adipose tissues increased by 434% (Figure 1C,D). However, significant changes were not observed in the weight gain of the gonadal adipose tissue following G-1 or G-36 administration (Figure 1D). On the last day of the experiment, we conducted an oral glucose tolerance test to determine obesity-induced glucose intolerance. The HFD-fed mice showed higher plasma glucose levels than the ND-fed mice (Figure 1E). Furthermore, G-1 administration reduced the peak increase in glucose levels, implying a normal capacity for glucose tolerance in mice (Figure 1E), whereas co-treatment with G-36 blocked this effect (Figure 1E). G-36 administration alone did not affect glucose intolerance (Figure 1E). Therefore, G-1 administration did not suppress HFD-induced increases in body weight gain and adiposity; however, it improved HFD-induced glucose intolerance in a GPER-dependent manner.

### 2.2. G-1 Suppressed Airway Hyperresponsiveness (AHR) and Immune Cell Infiltration in the Bronchoalveolar Lavage Fluid (BALF) of C57BL/6 Mice

**Figure 2:**
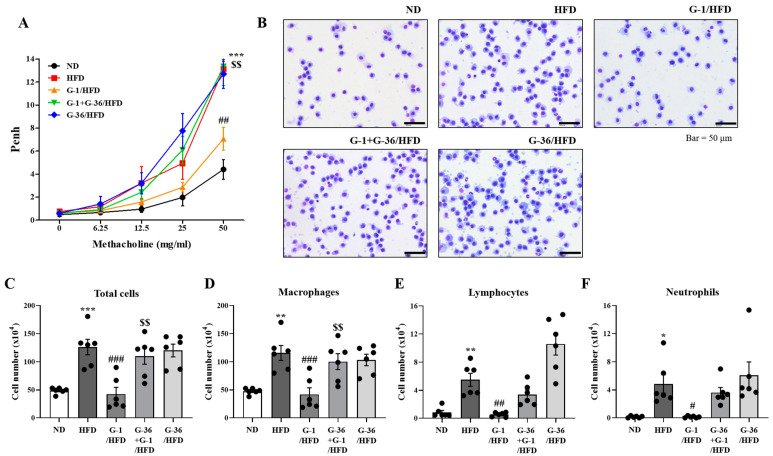
G-1 suppressed the HFD-induced AHR and infiltration of immune cells in the BALF of C57BL/6 mice. We assessed the effects of G-1 and/or G-36 on HFD-induced AHR and the infiltration of immune cells into the BALF of C57BL/6 mice. (**A**) We assessed AHR by whole-body plethysmography using increasing concentrations of methacholine. (**B**) We counted each cell type after May–Grünwald staining of immune cells in the BALF. (**C**) Total cell count. (**D**) Macrophage count. (**E**) Lymphocyte count. (**F**) Neutrophil count. ** *p* < 0.01, * *p* < 0.05, *** *p* < 0.001 vs. the ND group, ## *p* < 0.01, # *p* < 0.05 and ### *p* < 0.001 vs. the HFD group, $$ *p* < 0.01 vs. the G-1 group. Values are presented as the mean ± SEM of six mice.

To assess obesity-induced asthma, Penh values were measured to determine the effects of G-1 and G-36 on AHR. The HFD-fed obese mice showed significant increases in Penh values compared to ND-fed mice at a dose of 50 mg/mL methacholine, suggesting obesity-induced asthma (Figure 2A) [26,27]. G-1 administration significantly suppressed the increase in Penh values at a methacholine dose of 50 mg/mL, whereas co-treatment with G-36 blocked this effect (Figure 2A).

To check immune responses, we assessed immune cells in the BALF. The number of macrophages significantly increased in the HFD-fed mice (Figure 2B,D). G-1 administration reduced the number of macrophages, whereas co-treatment with G-36 blocked this effect (Figure 2B,D). G-36 administration alone did not affect the HFD-induced increase in macrophage numbers (Figure 2B,D). The total number of immune cells in the BALF showed similar changes: an increase by HFD feeding, blockage by G-1 administration, and disappearance of the G-1 effect by co-treatment with G-36 (Figure 2B,C). In addition to the increase in macrophage counts, the number of lymphocytes (Figure 2E) and neutrophils (Figure 2F) increased in the HFD-fed mice. G-1 administration significantly reduced the HFD-induced increase, whereas co-treatment with G-36 partially reversed this effect (Figure 2E,F). Therefore, G-1 treatment significantly inhibited the HFD-induced increase in immune cells in BALF in a GPER-dependent manner (Figure 2D–F). No eosinophils were observed, implying that HFD feeding resulted in obesity-induced asthma, which is distinct from eosinophil-induced allergic airway inflammation [27,28].

### 2.3. G-1 Suppressed Histopathological Changes in the Lungs of C57BL/6 Mice

**Figure 3:**
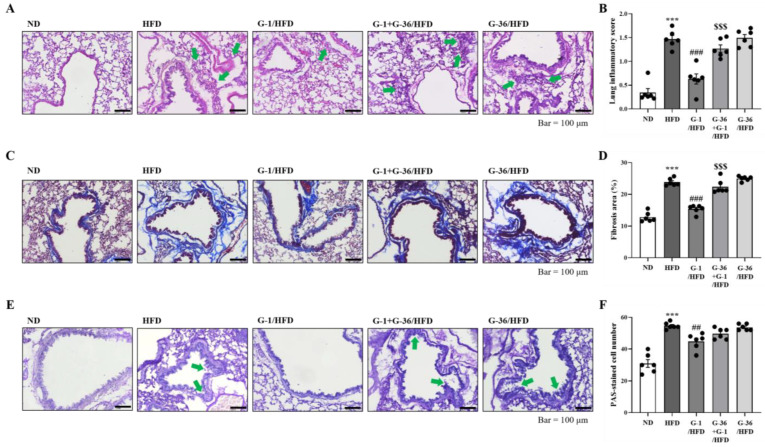
G-1 suppressed the HFD-induced histopathologic changes in the lungs of C57BL/6 mice. We assessed the effects of G-1 and/or G-36 on HFD-induced histopathological changes in the lungs. (**A**) Lung tissues were stained with H&E solution. Neutrophils were observed rarely in the ND group; however, HFD increased the number of neutrophils around the bronchioles, as indicated by the green arrows. G-1 administration suppressed this increase, which was reversed by co-treatment with G-36. (**B**) Histogram of the inflammation score. (**C**) Lung tissues were stained with MT solution. (**D**) Histogram of fibrous areas. (**E**) Lung tissues were stained with PAS solution. Purple-colored mucin staining was darker and denser in the HFD group than in the ND group, as indicated by the green arrows. G-1 administration made the mucin staining lighter and thinner, which was reversed by co-treatment with G-36. (**F**) Histogram of the PAS-stained cells. *** *p* < 0.001 vs. the ND group, ## *p* < 0.01, ### *p* < 0.001 vs. the HFD group, $$$ *p* < 0.001 vs. the G-1 group. Values are presented as the mean ± SEM of six mice.

We investigated airway inflammation induced by obesity using histopathological examination. Hematoxylin and eosin (H&E) staining of lung tissues showed few immune cells in ND-treated mice; however, high and dense accumulation of immune cells was observed in HFD-treated mice (Figure 3A). G-1 administration significantly suppressed HFD-induced accumulation, whereas cotreatment with G-36 reversed this effect (Figure 3A). We semi-quantitatively evaluated the degree of lung inflammation by measuring immune cell density around the bronchioles. The inflammation score in the ND group was approximately 0.35; however, HFD increased the score to approximately 1.45 (Figure 3B). G-1 administration significantly suppressed this effect to 0.6, whereas co-treatment with G-36 reversed this effect (Figure 3B). Masson’s trichrome (MT) staining showed narrow fibrotic areas stained blue in ND mice; however, HFD significantly increased the width and thickness of the blue-stained fibrotic areas (Figure 3C). G-1 administration significantly suppressed the fibrotic areas, whereas co-treatment with G-36 reversed this effect (Figure 3C,D). Periodic acid–Schiff (PAS) staining showed bright and thin areas of violet coloration around the bronchial airways in ND mice; however, the violet-colored areas were darker and thicker in HFD-fed mice, indicating mucus hypersecretion in the airways and hyperplasia of goblet cells (Figure 3E). G-1 administration significantly changed the violet-colored areas to brighter and thinner areas compared to those in HFD mice (Figure 3E). Co-treatment with G-36 reversed this effect; however, G-36 alone did not affect HFD-induced PAS staining (Figure 3E). We detected approximately 30 PAS-positive cells/mm in ND mice and approximately 55 PAS-positive cells/mm in HFD mice (Figure 3F).

### 2.4. G-1 Increased Anti-Inflammatory M2 Macrophages and Suppressed Innate Lymphoid Cells (ILCs) in the Lungs of C57BL/6 Mice

**Figure 4:**
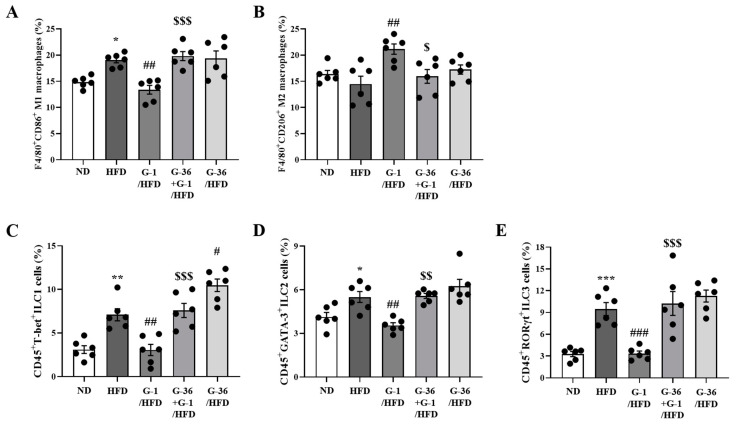
G-1 increased M2 macrophages and inhibited the HFD-induced rise of M1 macrophages and ILCs in the lungs of C57BL/6 mice. We assessed the effects of G-1 and/or G-36 on HFD-induced changes in M2 and M1 macrophages and ILC1, ILC2, and ILC3 in the lungs of C57BL/6 mice. (**A**) Percentages of F4/80^+^CD86^+^ M1. (**B**) F4/80^+^CD206^+^ M2 macrophages. (**C**) Percentage of CD45^+^FcεR1^-^T-bet^+^ ILC1 cells. (**D**) CD45^+^FcεR1^-^GATA-3^+^ ILC2 cells. (**E**) CD45^+^FcεR1^-^RORγt^+^ ILC3 cells. * *p* < 0.05, ** *p* < 0.01, and *** *p* < 0.001 vs. the ND group, # *p* < 0.05, ## *p* < 0.01 and ### *p* < 0.001 vs. the HFD-treated group, $ *p* < 0.05, $$ *p* < 0.01, $$$ *p* < 0.001 vs. the G-1 group. The results are presented as the mean ± SEM (*n* = 6).

Because obesity-induced asthma by HFD feeding is caused by chronic low-grade inflammation (metaflammation) in the adipose tissue, and major cells in the BALF were found to be macrophages, we determined the polarization of the macrophages. The proportions of classically activated pro-inflammatory M1 macrophages and alternatively activated anti-inflammatory M2 macrophages were assessed using flow cytometry of lung cells as F4/80^+^CD86^+^ and F4/80^+^CD206^+^ cells, respectively. As shown in Figure 4A, HFD significantly increased the population of M1 macrophages compared to that in ND-fed mice. G-1 administration significantly suppressed the HFD-induced increase in M1 macrophages, whereas co-treatment with G-36 reversed this effect (Figure 4A). In contrast, as shown in Figure 4B, HFD slightly decreased the population of M2 macrophages; however, G-1 administration significantly increased the proportion of M2 macrophages, which was reversed by co-treatment with G-36 (Figure 4B).

We also assessed the proportion of innate lymphoid cells (ILCs) in lungs. Generally, ILCs are categorized into three major groups: types 1, 2, and 3 ILCs (ILC1, ILC2, and ILC3) [29], which correspond to Th1, Th2, and Th17 cells, respectively [29]. Notably, ILC1 is an indicator of the severity of chronic obstructive pulmonary disease, ILC2 is a crucial player in allergic asthma, and ILC3 is found in neutrophilic asthma in respiratory diseases [29]. Figure 4C–E shows the population of ILC1, ILC2, and ILC3 cells, respectively. We found significantly HFD-induced increases in the population of CD45^+^FcεR1^-^T-bet^+^ ILC1, CD45^+^FcεR1^-^GATA-3^+^ ILC2, and CD45^+^FcεR1^-^RORγt^+^ ILC3, whereas G-1 administration suppressed the population of ILC1, ILC2, and ILC3 in the lungs. Co-treatment with G-36 reversed these effects of G-1 (Figure 4C–E). G-36 administration alone did not change the population of ILC2 and ILC3 in the lungs of HFD mice but significantly increased ILC1 cells (Figure 4C–E).

### 2.5. G-1 Increased Adiponectin Levels and Suppressed Leptin Levels in the Lungs and Gonadal White Adipose Tissue of C57BL/6 Mice

**Figure 5:**
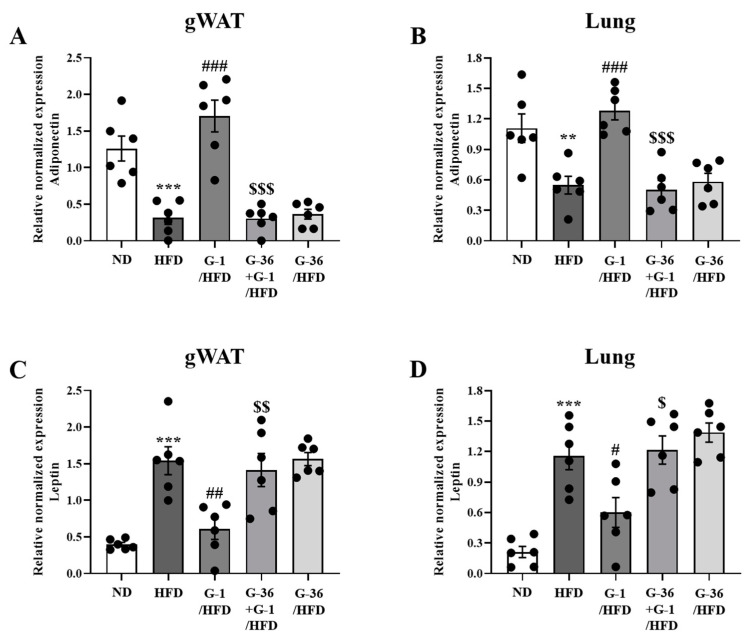
Effect of G-1 on HFD-induced modifications in adipokine levels in the gonadal adipose tissues and lungs of C57BL/6 mice. We assessed the effects of G-1 and/or G-36 on HFD-induced modifications in the levels of adipokines in the gonadal adipose tissue and lungs of C57BL/6 mice. mRNA expression of *Adipoq* (**A**,**B**) and *Lep* (**C**,**D**) in the gonadal white adipose tissue (**A**,**C**) and lungs (**B**,**D**) of mice. mRNA levels of adipokines were quantified as ratios relative to *Gapdh* mRNA levels. ** *p* < 0.01 and *** *p* < 0.001 vs. the ND group, # *p* < 0.05, ## *p* < 0.01, ### *p* < 0.001 vs. the HFD-treated group, $$ *p* < 0.01, $ *p* < 0.05, $$$ *p* < 0.001 vs. the G-1 group. Values represent the means ± SEMs (*n* = 6).

Obesity regulates the levels of adipokines such as adiponectin and leptin [30], and 17β-estradiol regulates the levels of adiponectin and leptin in adipocytes and adipose tissue [31,32]. We analyzed the levels of *Adipoq* and *Lep* in gonadal white adipose tissue and the lungs using qRT-PCR. Notably, *Adipoq* levels significantly decreased in the adipose tissue and lungs of HFD mice, whereas G-1 administration significantly reversed this decrease, which was reversed by co-treatment with G-36 (Figure 5A,B). Furthermore, *Lep* levels significantly increased in the adipose tissue and lungs of HFD mice, whereas G-1 administration significantly reversed this increase, which was reversed by co-treatment with G-36 (Figure 5C,D). G-36 administration alone did not alter the levels of *Adipoq* and *Lep* in HFD mice (Figure 5A,D).

### 2.6. G-1 Suppressed Inflammatory Cytokine Levels in the Gonadal White Adipose Tissue of C57BL/6 Mice

**Figure 6:**
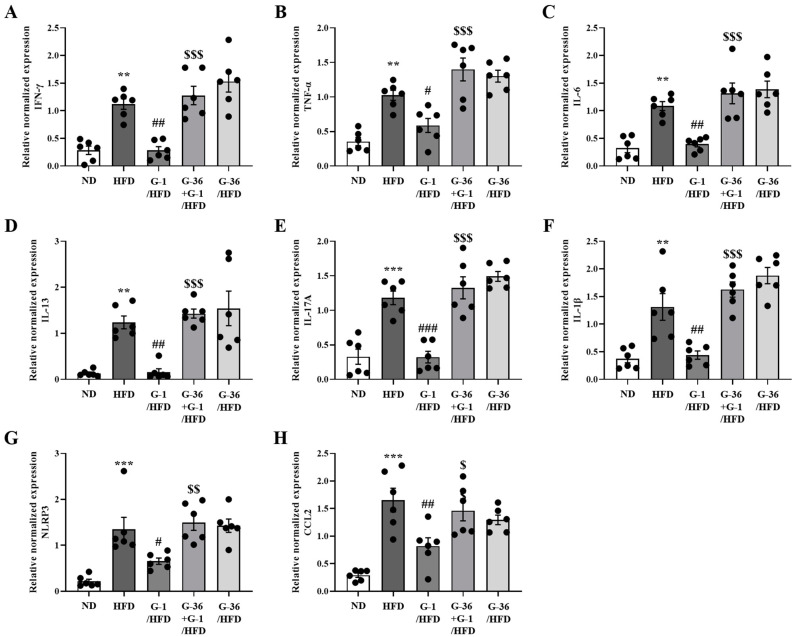
Effects of G-1 on the HFD-induced increase of pro-inflammatory cytokine levels in the gonadal adipose tissues of C57BL/6 mice. We assessed the effects of G-1 and/or G-36 on HFD-induced increase in pro-inflammatory cytokine levels in the gonadal adipose tissues of C57BL/6 mice. Normalized mRNA expression of (**A**) *Ifn-γ*, (**B**) *Tnf-α*, (**C**) *Il-6*, (**D**) *Il-13*, (**E**) *Il-17a*, (**F**) *Il-1β*, (**G**) *Nlrp-3*, and (**H**) *Ccl2* in gonadal white adipose tissue. We quantified the mRNA levels of cytokines as ratios relative to *Gapdh* mRNA levels. ** *p* < 0.01, *** *p* < 0.001 vs. the ND group, ### *p* < 0.001, ## *p* < 0.01, # *p* < 0.05 vs. the HFD-treated group, $$ *p* < 0.01, $ *p* < 0.05, $$$ *p* < 0.001 vs. the G-1 group. Values represent the means ± SEMs (*n* = 6).

Because chronic low-grade systemic inflammation in white adipose tissue may cause obesity-induced airway inflammation and asthma [33], we analyzed the levels of inflammatory cytokines and inflammation-related genes in gonadal white adipose tissue using qRT-PCR. Because multiple ILCs are present in the lungs [28,34], pro-inflammatory Th1 genes (*Ifn-γ*, *Tnf-α*, and *Il-6*), Th2 (*Il-13*), Th17 (*Il-17a*), inflammasome-related genes (*Il-1β* and *Nlrp3*), and a chemoattractant gene (*Ccl2*) were determined using qRT-PCR in gonadal white adipose tissue. The levels of *Inf-γ*, *Tnf-α*, *Il-6*, *Il-13*, *Il-17a*, *Il-1β*, *Nlrp3*, and *Ccl2* were significantly increased in the adipose tissue of HFD mice (Figure 6A–H), whereas G-1 administration significantly reduced this increase, which was reversed by co-treatment with G-36 (Figure 6A–H). G-36 administration alone did not change these levels in HFD mice (Figure 6A–H).

### 2.7. G-1 Suppressed Inflammatory Cytokine Levels in the Lungs of C57BL/6 Mice

**Figure 7:**
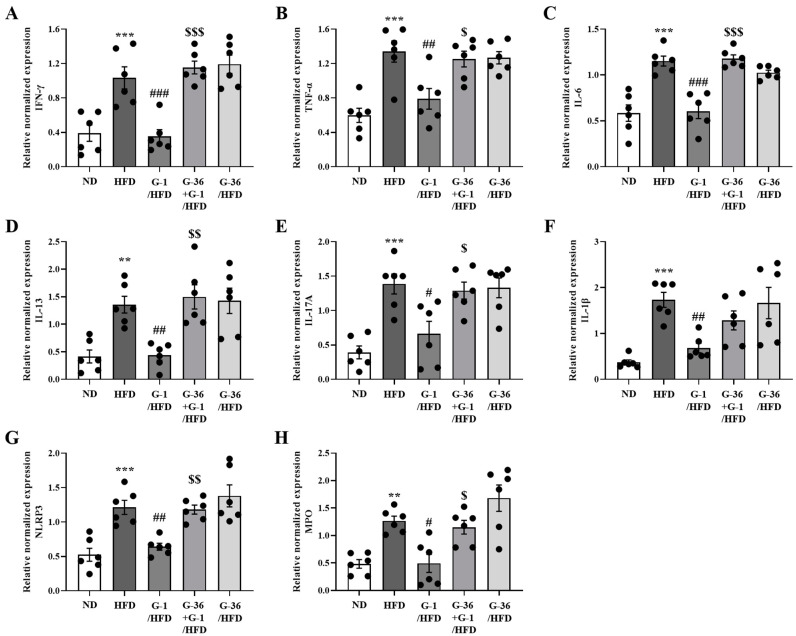
Effects of G-1 on the HFD-induced pro-inflammatory cytokine levels in the lungs of C57BL/6 mice. We assessed the effects of G-1 and/or G-36 on the HFD-induced increase in pro-inflammatory cytokine levels in the lungs of C57BL/6 mice. mRNA expression of (**A**) *Ifn-γ*, (**B**) *Tnf-α*, (**C**) *Il-6*, (**D**) *Il-13*, (**E**) *Il-17a*, (**F**) *Il-1β*, (**G**) *Nlrp-3*, and (**H**) *Mpo* in the lungs of mice. We quantified the mRNA levels of cytokines as ratios relative to *Gapdh* mRNA levels. ** *p* < 0.01, *** *p* < 0.001 vs. the ND group, # *p* < 0.05, ## *p* < 0.01, and ### *p* < 0.001 vs. the HFD-treated group, $$ *p* < 0.01, $ *p* < 0.05, $$$ *p* < 0.001 vs. the G-1 group. Values represent the means ± SEMs (*n* = 6).

We assessed multiple inflammation-related genes in the lungs using qRT-PCR, including pro-inflammatory Th1 genes (*Ifn-γ*, *Tnf-α*, and *Il-6*), Th2 (*Il-13*), Th17 (*Il-17a*), inflammasome-related genes (*Il-1β* and *Nlrp3*), and a neutrophil marker gene (*Mpo*) [28,34]. Pro-inflammatory gene levels increased significantly in the HFD group, whereas G-1 administration significantly suppressed this increase, which was reversed by cotreatment with G-36 (Figure 7A–H). G-36 administration alone did not change the levels of inflammation-related genes in HFD mice (Figure 7A–H).

### 2.8. G-1 Suppressed Inflammatory Cytokine Levels in the BALF of C57BL/6 Mice

**Figure 8:**
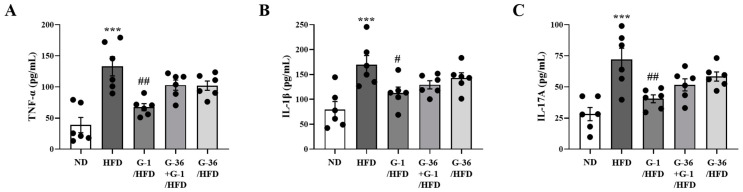
Effects of G-1 on the HFD-induced increase of pro-inflammatory cytokines in the BALF. We assessed the effects of G-1 and/or G-36 on the protein expression levels of (**A**) TNF-α, (**B**) IL-1β, and (**C**) IL-17A in the BALFs of C57BL/6 mice. We quantified protein levels using ELISA. *** *p* < 0.001 vs. the ND group, ## *p* < 0.01, # *p* < 0.05 vs. the HFD-treated group. Values represent the means ± SEMs (*n* = 6).

To confirm the changes in pro-inflammatory cytokine levels at the protein level, we measured the protein levels of TNF-α, IL-1β, and IL-17a in the BALF of the mice. Notably, HFD induced an increase in pro-inflammatory cytokines in the BALF of C57BL/6 mice (Figure 8A–C), whereas G-1 administration significantly suppressed this increase (Figure 8A–C).

## 3. Discussion

In the present study, for the first time, we found that G-1, a GPER-selective agonist, reduced HFD-induced AHR, airway inflammation in the lungs, and metaflammation in adipose tissue. Such a suppression by G-1 administration was blocked by co-treatment of G-36, a selective GPER antagonist, suggesting involvement of GPER in the action of G-1 in the suppression of obesity-induced asthma.

Although a cerebroventricular injection of G-1 has been reported to induce an anorectic response in hypothalamus [35], no change in food intake was observed in GPER-deficient mice [24]. We were unable to observe changes in body weight. Additionally, GPER-dependent suppressive effects of estrogen on adipogenesis have been reported [36]. Furthermore, G-1 attenuated triglyceride accumulation and fatty acid synthesis in human and rodent pancreatic islets and β cells [37]. However, in the present study, change was not observed in the HFD-induced adiposity following G-1 administration, which is consistent with no significant increases in body fat content observed in GPER-deficient mice [24]. We found that G-1 improved glucose intolerance, which was supported by the observation that G-1 treatment stimulated insulin secretion from islets in a GPER-dependent manner [38].

G-1 suppression of the expression levels of pro-inflammatory cytokines, leptin, and the pro-inflammatory M1 macrophage population in the lungs may mainly contribute to its in vivo efficacy. In addition, G-1 induction of the expression levels of anti-inflammatory adiponectin and the population of anti-inflammatory M2 macrophages also contributed to the anti-asthmatic effects. How can G-1 activation of GPER lead to switching of the M1 to M2 macrophage phenotype and reduce metaflammation? Estrogen has been reported to suppress production of IL-2 and IFN-γ and increase IL-10 expression in the draining lymph node cells [39]. In addition, estrogen treatment decreases the delayed-type hypersensitivity response, downregulates the functions of antigen-presenting cells, and switches the Th1 immune response to a Th2 immune response [39]. These observations suggest that G-1 activation in immune cells leads to an anti-inflammatory state, resulting in immune suppression. Forkhead box P3 (FoxP3) protein plays a prominent role in the immunomodulatory effects of GPER activation by enhancing IL-10 secretion and regulatory T cell differentiation [40,41]. Although further investigation is required to understand the precise mechanism by which GPER activation leads to immunosuppression through FoxP3-dependent regulatory T cells, we provide evidence to support GPER-mediated immunosuppression: (1) switching M1 to M2 macrophages; (2) suppression of ILC 1, 2, and 3 populations in the lungs; (3) suppression of pro-inflammatory cytokines and leptin expression in the gonadal adipose tissue and lungs; and (4) increase of anti-inflammatory adiponectin expression in the gonadal adipose tissue and lungs.

IL-13 is a typical cytokine associated with promoting M2 macrophage polarization, anti-inflammatory responses, and tissue repair function [42]. However, we observed the reduced expression of IL-13 after administration of G-1. Then, how G-1 activation of GPER could induce a shift from M1 to M2 macrophage polarization in concomitant reduction of IL-13 expression. Two mechanisms could be considered. First, the increase in IL-13 has been observed in certain contexts, such as obesity or chronic inflammation, probably due to a combination of increased activity of ILC2s and Th2 cells within inflamed adipose tissue [43]. Then, the reduction of IL-13 levels in the present study may diminish the M1 polarization driving stimuli, which consequently facilitates a shift toward M2 macrophage polarization, promotes an anti-inflammatory environment, and aids tissue repair. Second, GPER activation may directly promote the shift to M2 macrophages through the AMP-activated protein kinase (AMPK) pathway [44]. AMPK plays a crucial role in energy sensing and metabolic regulation [45]. GPER activation by G-1 could lead to metabolic reprogramming via AMPK, which is essential for the M2 phenotype. While M1 macrophages primarily rely on glycolysis for energy, M2 macrophages depend on oxidative phosphorylation [46]. G-1 activation of GPER may enhance the shift from M1 to M2 by promoting this metabolic transition through AMPK. This mechanism supports the idea that even with reduced IL-13 levels, G-1 activation of GPER can still drive the transition from M1 to M2 macrophages by facilitating the necessary metabolic changes, thus resulting in an anti-inflammatory state.

The present GPER-mediated in vivo effects in obesity-induced asthma are correlated not only with the above-mentioned in vitro cell-level immunosuppressive actions of GPER but also with several in vivo immunosuppressive observations of the GPER gene. For instance, in aged GPER-deficient male mice, the inhibitory function of GPER in immune responses was demonstrated by an enhanced pro-inflammatory state [23]. In GPER-deficient mice, inflammation exacerbation has been observed in multiple sclerosis models [47,48]. In postmenopausal mice, G-1 treatment effectively reduces vascular inflammation and atherosclerosis [49]. Therefore, using G-1 and G-36, we provided strong evidence that GPER could be a good therapeutic target for inflammatory diseases, including obesity-induced asthma, by regulating immune responses.

In our study, the macrophage phenotype observed in the HFD-induced obese asthma model predominantly exhibited M1 macrophage characteristics. This phenotype is typically associated with pro-inflammatory responses and is thought to play a central role in the pathogenesis of obesity-related asthma [50]. The chronic low-grade inflammation observed in obesity is a key driver of this M1 polarization [51]. Moreover, the shift towards M1 macrophage dominance is further driven by the altered adipokine profile in obesity, particularly the elevated levels of leptin and reduced levels of adiponectin, both of which influence macrophage polarization [52,53]. These findings highlight the critical role of M1 macrophages in the progression of obesity-induced asthma, where they contribute to both systemic inflammation and airway hyperresponsiveness. Targeting the inflammatory pathways that promote M1 macrophage polarization may therefore represent a potential therapeutic strategy for managing asthma in obese individuals.

Limitations: Although pharmacological tools of G-1 and G-36 support the involvement of GPER, utilization of GPER gene deficient mice may strengthen the present results. In the present study, male mice were utilized. It would be favorable to expand the study with female mice in combination with ovarectomy-induced estrogen loss. Through in vitro studies, it should be determined not only how GPER activation leads macrophages to M2 phenotypes and suppresses M1 phenotypes but also how GPER activation could lead T cells into FoxP3 positive regulatory T cells.

## 4. Materials and Methods

### 4.1. Materials

G-1 and G-36 were obtained from Cayman Chemical Co. (Ann Arbor, MI, USA). A high-fat rodent diet (60 kcal% fat, cat. D12492) was acquired from Research Diets (New Brunswick, NJ, USA). Other chemicals were sourced from Sigma-Aldrich (St. Louis, MO, USA).

### 4.2. Mouse Strain

C57BL/6 mice, obtained from DBL Company (Seoul, Republic of Korea), were maintained in the laboratory animal facility at Kyung Hee University. They were given free access to standard chow and water. Environmental conditions were strictly controlled, with temperatures set between 22 and 24 °C, humidity maintained at 60 ± 5%, and a light/dark cycle of 12 h (lights on from 7:00 AM to 7:00 PM). The mice were housed in pairs within standard plastic cages containing sawdust bedding. The animal protocol was reviewed and approved by the Institutional Animal Care and Use Committee of Kyung Hee University (KHSASP-24-058) on 14 February 2024, ensuring ethical treatment and care of the animals.

### 4.3. HFD Feeding

We randomly assigned six-week-old mice to five groups. We fed control C57BL/6 mice (*n* = 6) with a standard chow diet (ND) for 15 weeks, while HFD C57BL/6 mice (*n* = 6) had a diet containing 60% (*w*/*w*) fat (HFD, Research Diets, New Brunswick, NJ, USA) for the same duration (Figure 1A). We administered G-1 or G-36 (0.5 mg/kg, intraperitoneally) five days per week to mice in the HFD + G-1 and HFD + G-36 groups during the last 5 weeks (*n* = 6 for each group) (Figure 1A). Mice in the HFD + G-1 + G-36 group were given both G-1 and G-36 (each at 0.5 mg/kg, intraperitoneally) five times a week during the final 5 weeks (*n* = 6). As we wanted to isolate the impact of obesity and the associated metabolic alterations on airway function, mice were not exposed to additional sensitizing agents like house dust mite or ovalbumin.

### 4.4. Oral Glucose Tolerance Test (OGTT)

During the final week, G-1 and/or G-36 were administered on weekdays. We performed an oral glucose tolerance test (OGTT) on the Saturday, followed by airway hyperresponsiveness (AHR) measurements on the Sunday. Prior to the OGTT, the mice were fasted for 6 h. We gave D-(+)-glucose at a dose of 2 g/kg body weight orally. We measured the levels of blood glucose from the tail with strips (Handok, Seoul, Republic of Korea) and Barozen II glucometers at baseline and at the indicated times.

### 4.5. Airway Hyperresponsiveness (AHR) to Methacholine

Airway hyperresponsiveness (AHR) was assessed using a non-invasive lung function measurement device, Model PLY-UNR-MS2 (EMKA Technologies, Paris, France). We placed mice in a barometric plethysmographic chamber, and recorded baseline measurements for 3 min. We calculated enhanced pause (Penh) as per the manufacturer’s instructions. The results are presented as the percentage increase in Penh in response to escalating concentrations of methacholine [54].

### 4.6. Bronchial Alveolar Lavage Fluid (BALF) Cell Enumeration and Analysis

We collected immune cells in the BALF in 0.4 mL phosphate-buffered saline (PBS). A 20 μL of Wright’s solution and a 20 μL aliquot of the sample were combined, and the 40 μL mixture was then loaded into a hemocytometer. We counted the cells, and calculated the total number by multiplication. For each type of immune cells, we centrifuged 50 μL of the BALF cell suspension onto a glass slide using a Cellspin^®^ centrifuge (Hanil Electric, Seoul, Republic of Korea), fixed the cells in methanol for 30 s, and stained with May–Grünwald solution for 8 min and subsequently Giemsa solution for 12 min. We identified cells as macrophages, lymphocytes, or neutrophils based on morphological features and staining. For each sample, we counted 500 cells to calculate the percentage of each cell type.

### 4.7. Histological Examination of Lung Tissue

Mice were sacrificed by cervical dislocation following carbon dioxide gas exposure (concentration: 60–70%). We stained lung tissue sections with periodic acid–Schiff (PAS), Masson’s trichrome (MT), and hematoxylin and eosin (H&E) to determine mucus-producing cells, fibrosis, and infiltration of immune cells, respectively [54]. We evaluated the levels of lung inflammation on a subjective scale of 0 to 3 by an observer blinded to the treatment groups. When no inflammation was present, a score of 0 was assigned, occasional inflammation and cuffing for 1, a thin layer (one to five cells thick) of inflammatory cells surrounding most bronchi or vessels for 2, and a thick layer (more than five cells thick) around the majority of bronchi or vessels for 3.

### 4.8. Quantitative Real-Time PCR (qRT-PCR)

We synthesized first-strand cDNA from total RNA isolated with TRIzol reagent (Invitrogen, Waltham, MA, USA) in order to assess the levels of inflammatory markers expression in mouse tissues. The cDNA along with gene-specific primers and Promega Go-Taq DNA polymerase (Madison, WI, USA) were used for PCR. We conducted quantitative PCR utilizing Thunderbird Next SYBR qPCR Mix (Toyobo, Osaka, Japan) on a CFX Connect Real-Time System (Bio-Rad, Hercules, CA, USA). The conditions for PCR were as follows: one cycle at 95 °C for 4 min, followed by 40 cycles of 95 °C for 30 s and 57 °C for 30 s, and a final cycle at 95 °C for 30 s. Gene expression was normalized to Gapdh levels. Primer sequences for the cytokines are listed in Table 1.

### 4.9. Flow Cytometry

To measure M1 and M2 macrophage populations, single cells isolated from the lungs were first stained with anti-CD206 (cat. 17-2061-82, eBioscience, San Diego, CA, USA) or APC-conjugated rat anti-CD86 (cat. 17-0862-81, eBioscience) and FITC-conjugated rat anti-F4/80 antibody (cat. 11-4801-81, eBioscience) at 4 °C for 45 min. After staining, cells were fixed with IC Fixation Buffer (cat. 00-8222-49, eBioscience) at room temperature for 1 h.

For the analysis of innate lymphoid cells (ILCs), including group 3 ILCs (CD45^+^ FcεR1^-^ RORγt^+^), group 2 ILCs (CD45^+^ FcεR1^-^ GATA-3^+^), and group 1 ILCs (CD45^+^ FcεR1^-^ T-bet^+^), single cells from the lungs were initially stained with FITC-conjugated Armenian hamster anti-FcεR1 (cat. 11-5898-81, eBioscience) and eFluor 450-conjugated rat anti-CD45 (cat. 48-0451-80, eBioscience) at 4 °C for 45 min. Following fixation with IC Fixation Buffer (cat. 00-8222-49, eBioscience) at room temperature for 1 h, cells were permeabilized and subsequently stained at room temperature for 1 h with APC-conjugated rat anti-RORγt (cat. 17-6988-82, eBioscience), rat anti-GATA-3 (cat. 50-9966-41, eBioscience), and oreFluor 660-conjugated mouse anti-T-bet (cat. 50-5825-82, eBioscience). We employed a CytoFLEX Flow Cytometer (Beckman Coulter, Brea, CA, USA) for cell sorting.

### 4.10. Enzyme-Linked Immunosorbent Assay (ELISA)

Levels of TNF-α, IL-1β, and IL-17A in the BALF of mice were measured using ELISA kits. Specific capture and biotinylated detection antibodies for TNF-α (cat. 88-7324-88), IL-1β (cat. BMS6002TWO), and IL-17A (cat. 88-7371-88) were sourced from eBioscience (San Diego, CA, USA). The assays utilized avidin–horseradish peroxidase (HRP) and absorbance was read at 450 nm.

### 4.11. Statistics

We presented all data as mean ± SEM (*n* = 6). We used one-way ANOVA to assess the treatment effect, followed by Tukey’s multiple comparison test. We analyzed statistical significance using GraphPad Prism version 5 (GraphPad, San Diego, CA, USA). We set statistical significance at *p* < 0.05. * *p* < 0.05, ** *p* < 0.01, and *** *p* < 0.001 vs. the ND group, # *p* < 0.05, ## *p* < 0.01, and ### *p* < 0.001, vs. the HFD-treated group, $ *p* < 0.05, $$ *p* < 0.01, and $$$ *p* < 0.001, vs. the G-1 group.

## Figures and Tables

**Table 1:** Primer sequences.

Mouse Gene	Forward Sequence (5′ to 3′)	Reverse Sequence (5′ to 3′)
*Il-4*	CCTCACAGCAACGAAGAACA	CTGCAGCTCCATGAGAACAC
*Il-5*	CACCAGCTATGCATTGGAGA	CTCTTTTTGGCGGTCAATGT
*Il-6*	CTGATGCTGGTGACAACCAC	TCCACGATTACCCAGAGAAC
*Il-13*	CAGCATGGTATGGAGTGTGG	AGGCCATGCAATATCCTCTG
*Il-17a*	AGCTGGACCACCACATGAAT	AGCATCTTCTCGACCCTGAA
*Ifn-γ*	CACGGCACAGTCATTGAAAG	GTCACCATCCTTTTGCCAGT
*Il-22*	CCGAGGAGTCAGTGCTAAGG	CATGTAGGGCTGGAACCTGT
*Il-23*	GACCCACAAGGACTCAAGGA	CATGGGGCTATCAGGGAGTA
*Il-1β*	CAGGCAGGCAGTATCACTCA	TGTCCTCATCCTGGAAGGTC
*Tnf-α*	ACGGCATGGATCTCAAAGAC	AGATAGCAAATCGGCTGACG
*Mpo*	CACTGGACACTGCAACAACA	CCATTGCGATTGACTCCAGG
*Nlrp3*	ATGCTGCTTCGACATCTCCT	GTTTCTGGAGGTTGCAGAGC
*Ccl2*	TGAATGTGAAGTTGACCCGT	ACAGAAGTGCTTGAGGTGGT
*Leptin*	TTCCTGTGGCTTTGGTCCTA	CGACTGCGTGTGTGAAATGT
*Adipoq*	TACTGCAACATTCCGGGACT	GTAGGTGAAGAGAACGGCCT
*Gapdh*	AACTTTGGCATTGTGGAAGG	GGATGCAGGGATGATGTTCT

## Data Availability

Data are available from the authors upon request.
